# Supplementary material for: Incoherent scattering can favorably influence energy filtering in nanostructured thermoelectrics
Source: Sci Rep. 2017 Aug 11;7:7879. doi: 10.1038/s41598-017-07935-w (PMC5554188; doi:10.1038/s41598-017-07935-w)
Supplement: Supplementary file 1 — Incoherent scattering can favorably influence energy filtering in nanostructured thermoelectrics [file 41598_2017_7935_MOESM1_ESM.pdf]

Incoherent scattering can favorably influence energy filtering in nanostructured thermoelectrics

(Supplementary Material)

Aniket Singha[1,*] and Bhaskaran Muralidharan[1]

[1]*Department of Electrical Engineering,*

Indian Institute of Technology Bombay, Powai, Mumbai-400076, India

(Dated: June 12, 2017)

Abstract

This material contains the details of the methods used for simulation of our results as well as the derivation of the relevant parameters used to justify our discussions. It also contains some results for the different current profiles in case of higher order scattering mechanisms.

* aniketsingha@ee.iitb.ac.in

I. NEGF EQUATIONS FOR ELECTRONIC TRANSPORT MEDIATED BY INCOHERENT SCATTERING ALONG WITH INTERMODE COUPLING

In case of non-dissipative transport in nano devices, the generalized equations of the Green's function and scattering matrices for the mth mode are given by [2–4]

$$\begin{aligned} G(\overrightarrow{k_m}, E) &= [EI - H - U - E_m - \Sigma(\overrightarrow{k_m}, E)]^{-1} \\ \Sigma(\overrightarrow{k_m}, E) &= \Sigma_L(\overrightarrow{k_m}, E) + \Sigma_R(\overrightarrow{k_m}, E) + \Sigma_s(\overrightarrow{k_m}, E) \\ A(\overrightarrow{k_m}, E) &= i[G(\overrightarrow{k_m}, E) - G^\dagger(\overrightarrow{k_m}, E)] \\ \Gamma(\overrightarrow{k_m}, E) &= [\Sigma(\overrightarrow{k_m}, E) - \Sigma^\dagger(\overrightarrow{k_m}, E)], \end{aligned} \tag{1}$$

where H is the discretized Hamiltonian matrix (constructed using the effective mass approach [3]), $U = -eV$ is the electronic potential energy in the band and $E_m = \frac{\hbar^2 k_m^2}{2m_t}$ is the sub-band energy due to momentum along the transverse direction. $\Sigma_L(\overrightarrow{k_m}, E) + \Sigma_R(\overrightarrow{k_m}, E)$ and $\Sigma_s(\overrightarrow{k_m}, E)$ describe the effect of coupling and scattering of electronic wave functions due to contacts and electron-phonon interaction respectively. The device Hamiltonian, constructed using the effective mass approach[2–4].

In the above sets of equations, $\overrightarrow{k_m}$ denote the transverse wave-vector and E is the free variable denoting the energy of the electronic wave function. $A(\overrightarrow{k_m}, E)$ is the $1-D$ spectral function for the mth sub-band and $\Gamma(\overrightarrow{k_m}, E)$ is the broadening matrix for the mth sub-band at energy E. We assume that both the contacts are symmetrically coupled and that the potential drop is linear across the energy barriers. Assuming quasi-equilibrium electronic distribution at the contacts, their respective electrochemical potentials are given by $\mu_{H/C} = \mu_0 \pm V/2$. One can then say that V is the potential drop across the thermoelectric generator due to current flow through an external passive circuit element. For moderate electron-phonon interaction, it is generally assumed that the real part of $\Sigma_s = 0$. Hence,

$$\Sigma_s(\overrightarrow{k_m}, E) = i\frac{\Gamma_s(\overrightarrow{k_m}, E)}{2} = i\frac{\Sigma_s^{in}(\overrightarrow{k_m}, E) + \Sigma_s^{out}(\overrightarrow{k_m}, E)}{2} \tag{2}$$

$\Sigma^{in}(\overrightarrow{k_m}, E)$ and $\Sigma^{out}(\overrightarrow{k_m}, E)$ are the in-scattering and the out-scattering functions which models the rate of scattering of the electrons from the contact and inside the device.

$$\Sigma^{in}(\overrightarrow{k_m}, E) = \Sigma_L^{in}(\overrightarrow{k_m}, E) + \Sigma_R^{in}(\overrightarrow{k_m}, E) + \Sigma_s^{in}(\overrightarrow{k_m}, E)$$

$$\Sigma^{out}(\overrightarrow{k_m}, E) = \Sigma_L^{out}(\overrightarrow{k_m}, E) + \Sigma_R^{out}(\overrightarrow{k_m}, E) + \Sigma_s^{out}(\overrightarrow{k_m}, E), \tag{3}$$

where the subscript $'L'$, $'R'$ and $'s'$ denote the influence of left contact, right contact and electron-phonon scattering respectively. The in-scattering and out-scattering functions are dependent on the contact quasi-Fermi distribution functions as:

$$\Sigma^{in}(\overrightarrow{k_m}, E) = \underbrace{\Gamma_L(\overrightarrow{k_m}, E)f_L(E)}_{inflow\ from\ left\ contact} + \underbrace{\Gamma_R(\overrightarrow{k_m}, E)f_R(E)}_{inflow\ from\ right\ contact} + \underbrace{\Sigma_s^{in}(\overrightarrow{k_m}, E)}_{inflow\ due\ to\ phonons}$$
$$\Sigma^{out}(\overrightarrow{k_m}, E) = \underbrace{\Gamma_L(\overrightarrow{k_m}, E)\Big\{1 - f_L(E)\Big\}}_{outflow\ to\ left\ contact} + \underbrace{\Gamma_R(\overrightarrow{k_m}, E)\Big\{1 - f_R(E)\Big\}}_{outflow\ to\ right\ contact} + \underbrace{\Sigma_s^{out}(\overrightarrow{k_m}, E)}_{outflow\ due\ to\ phonons}, \tag{4}$$

where $f_{L(R)}$ denote the quasi-Fermi distribution of left(right) contact. The rate of scattering of electrons due to phonons is dependent on the electron and the hole correlation functions (G^n and G^p) and is given by:

$$\Sigma_s^{in}(\overrightarrow{k_m}, E) = diag\left[D_O \sum_{\overrightarrow{q_t}} G^n(\overrightarrow{k_m} + \overrightarrow{q_t}, E)\right]$$
$$\Sigma_s^{out}(\overrightarrow{k_m}, E) = diag\left[D_O \sum_{\overrightarrow{q_t}} G^p(\overrightarrow{k_m} + \overrightarrow{q_t}, E)\right]. \tag{5}$$

$G^n(\overrightarrow{k_m}, E)$ and $G^p(\overrightarrow{k_m}, E)$ are the electron and the hole correlation functions for the mth sub-band and $\{\overrightarrow{q_t}\}$ denotes the set of transverse phonon wave vectors. The electron and the hole correlation functions are again related to the electron in-scattering and the electron out-scattering functions via the equations:

$$G^n(\overrightarrow{k_m}, E) = G(\overrightarrow{k_m}, E)\Sigma^{in}(\overrightarrow{k_m}, E)G^{\dagger}(\overrightarrow{k_m}, E)$$
$$G^p(\overrightarrow{k_m}, E) = G(\overrightarrow{k_m}, E)\Sigma^{out}(\overrightarrow{k_m}, E)G^{\dagger}(\overrightarrow{k_m}, E) \tag{6}$$

Solving the dynamics of the entire system involves a self consistent solution of (1), (2), (5) and (6). For momentum scattering due to acoustic phonons, D_O in the above equations can be related to the acoustic phonon deformation potential (D_{ac}) by:

$$D_O = \frac{D_{ac}^2 k_B T F}{\rho v_s^2 a^3}, \tag{7}$$

where F is known as the form factor and denotes the spacial spread of the phonon wave-vectors. ρ and v_s denote the mass density and the velocity of sound in the material respectively. For the purpose of our simulation, we have used the parameters of bulk silicon. The spectral function for the mth sub-band is given by

$$A(\overrightarrow{k_m}, E) = G^n(\overrightarrow{k_m}, E) + G^p(\overrightarrow{k_m}, E),$$

while the electron density at the grid point j can be calculated from the above equations as:

$$n_j = \sum_m \int \frac{[G^n(\overrightarrow{k_m}, E)dE]}{\pi a A}$$

The intermode coupling current can be calculated from the phonon scattering matrices using the formula,

$$I_{Intermode} = \frac{2e}{h} \sum_{N_h} \sum_{\overrightarrow{k_m}} \int \Sigma_s^{out}(\overrightarrow{k_m}, E) G^n(\overrightarrow{k_m}, E) - \Sigma_s^{in}(\overrightarrow{k_m}, E) G^p(\overrightarrow{k_m}, E) dE, \tag{8}$$

where N_h is the number of lattice points between the hot contact and the barrier interface.

II. DERIVATION OF SCATTERING SELF-ENERGIES FOR HIGHER ORDER LOCAL SCATTERING MECHANISMS.

For elastic and local scattering mechanisms, the rate of electron scattering due to phonons in a semi-classical approximation is given by: [5, 6]:

$$\frac{\partial f(r, \overrightarrow{k}, t)}{\partial t} = \sum_{\overrightarrow{k'}} \Big\{ \underbrace{S(\overrightarrow{k'}, \overrightarrow{k})\{1 - f(r, \overrightarrow{k}, t)\} f(r, \overrightarrow{k'}, t)}_{in-scattering} - \underbrace{S(\overrightarrow{k}, \overrightarrow{k'})\{1 - f(r, \overrightarrow{k'}, t)\} f(r, \overrightarrow{k}, t)}_{out-scattering} \Big\} \delta(E_k - E_{k'}) \tag{9}$$

$S(\overrightarrow{k}, \overrightarrow{k'})/S(\overrightarrow{k'}, \overrightarrow{k})$ incorporate the dependence of the rate of electron scattering on energy/momentum. For isotropic scattering with acoustic phonons, $S(\overrightarrow{k'}, \overrightarrow{k})$ is independent of $\overrightarrow{k'}$ or $\overrightarrow{k}$

$$S(\overrightarrow{k'}, \overrightarrow{k}) = S(\overrightarrow{k}, \overrightarrow{k'}) = S(E_{\overrightarrow{k}}) = S(E_{\overrightarrow{k'}}) = \frac{2\pi k_B T D_{ac}^2}{\rho \hbar v_s^2 A}, \tag{10}$$

where D_{ac}, ρ and v_s are the acoustic deformation potential, the mass density and the velocity of sound in the medium respectively [5, 6]. The right side of Eq. 9 can be simplified by summing over the states (assuming steady state) [5, 6]:

$$\begin{aligned}\frac{\partial f(r,\overrightarrow{k},t)}{\partial t} &= \{1-f(r,\overrightarrow{k})\}\sum_{\overrightarrow{k'}} S(\overrightarrow{k'},\overrightarrow{k})f(r,\overrightarrow{k'})\delta(E_k-E_{k'}) \\ &\quad -f(r,\overrightarrow{k})\sum_{\overrightarrow{k'}} S(\overrightarrow{k},\overrightarrow{k'})\{1-f(r,\overrightarrow{k'})\}\delta(E_k-E_{k'}) \\ &= \{1-f(r,\overrightarrow{k})\}S(E_{\overrightarrow{k}})\sum_{\overrightarrow{k'}} f(r,\overrightarrow{k'})\delta(E_k-E_{k'}) \\ &\quad -f(r,\overrightarrow{k})S(E_{\overrightarrow{k}})\sum_{\overrightarrow{k'}}\{1-f(r,\overrightarrow{k'})\}\delta(E_k-E_{k'}) \end{aligned} \tag{11}$$

$$\Rightarrow \frac{\partial f(r,\overrightarrow{k})}{\partial t} = \{1-f(r,\overrightarrow{k})\}\underbrace{S(E_{\overrightarrow{k}})n_{tot}(r,E_{\overrightarrow{k}})}_{\frac{2\pi}{\hbar}\Sigma^{in}(E_{\overrightarrow{k}})} - f(r,\overrightarrow{k})\underbrace{S(E_{\overrightarrow{k}})p_{tot}(r,E_{\overrightarrow{k}})}_{\frac{2\pi}{\hbar}\Sigma^{out}(E_{\overrightarrow{k}})}, \tag{12}$$

where

$$\begin{aligned} n_{tot}(r,E_{\overrightarrow{k}}) &= \sum_{\overrightarrow{k'}} n(r,E_{\overrightarrow{k'}})\delta(E_{\overrightarrow{k}}-E_{\overrightarrow{k'}}) \\ p_{tot}(r,E_{\overrightarrow{k}}) &= \sum_{\overrightarrow{k'}} p(r,E_{\overrightarrow{k'}})\delta(E_{\overrightarrow{k}}-E_{\overrightarrow{k'}}) \end{aligned}$$

For acoustic phonon, $S(E_{\overrightarrow{k}})$ is independent of $E_{\overrightarrow{k}}$, $n_{tot}(E_{\overrightarrow{k}}) \approx D(E)f(r,E)$ and $p_{tot}(E_{\overrightarrow{k}}) \approx D(E)\{1-f(r,E)\}$. Therefore,

$$\begin{aligned} \tau(E_{\overrightarrow{k}}) &\propto \frac{1}{\frac{\partial f(r,\overrightarrow{k})}{\partial t}} \propto \frac{1}{D(E)} \\ &\Rightarrow \tau(E_{\overrightarrow{k}}) \propto E^{n}_{\overrightarrow{k}}, \end{aligned} \tag{13}$$

where $n = 0.5,\ 0,\ -0.5$ for $1-D,\ 2-D$ and $3-D$ devices respectively. To demonstrate the effect of the scattering which are of order higher than phonon scattering, we choose

$$S(E_{\overrightarrow{k}}) = kE^{-u}_{\overrightarrow{k}},$$

where k is a constant of proportionality such that

$$\tau(E_{\overrightarrow{k}}) \propto E^{n+u}_{\overrightarrow{k}},$$

where n is same as defined above and $r = -(n+u)$ being the order of the scattering process. In NEGF, we then use

$$\Sigma_s^{in}(r, E_{\overrightarrow{k}}) = \frac{\hbar}{2\pi} S(E_{\overrightarrow{k}}) \sum_{\overrightarrow{k'}} n(r, E_{\overrightarrow{k'}}) \delta(E_{\overrightarrow{k}} - E_{\overrightarrow{k'}})$$
$$\Sigma_s^{out}(r, E_{\overrightarrow{k}}) = \frac{\hbar}{2\pi} S(E_{\overrightarrow{k}}) \sum_{\overrightarrow{k'}} p(r, E_{\overrightarrow{k'}}) \delta(E_{\overrightarrow{k}} - E_{\overrightarrow{k'}}) \tag{14}$$

III. DERIVATION OF THE FACTOR Υ

In case of diffusive or incoherent transport without externally applied magnetic field, the dynamics of the electron system follows the quasi-distribution function given by [1]

$$f(\overrightarrow{k}) = f_0(E_{\overrightarrow{k}}) + \int_0^\infty P(\overrightarrow{k}, \tau') \Big\{ (-\frac{\partial f_0}{\partial E}) \overrightarrow{v}(\overrightarrow{k}). \Big(-e\overrightarrow{\mathcal{E}} - \nabla\mu - \frac{E-\mu}{T}\nabla T \Big) \Big\} d\tau',$$

where $P(\overrightarrow{k}, \tau')$ is the fraction of the electrons with wave vector $\overrightarrow{k}$ that donot suffer a scattering within the time period τ'. For isotropic scattering, generally $P(\overrightarrow{k}, \tau')$ takes the form [1]:

$$P(\overrightarrow{k}, \tau') = e^{\frac{-\tau'}{\tau(\overrightarrow{k})}} \tag{15}$$

Generally for isotropic and local scattering processes, $\tau(\overrightarrow{k})$ depends on $\overrightarrow{k}$ through the energy $E_{\overrightarrow{k}}$. Therefore,

$$P(\overrightarrow{k}, \tau') = e^{-\tau'/\tau(E_{\overrightarrow{k}})} \tag{16}$$

Equation (15) then becomes

$$f(\overrightarrow{k}) = f_0(E_{\overrightarrow{k}}) + \int_0^\infty e^{-\tau'/\tau(E_{\overrightarrow{k}})} \Big\{ (-\frac{\partial f_0}{\partial E}) \overrightarrow{v}(\overrightarrow{k}). \Big(-e\overrightarrow{\mathcal{E}} - \nabla\mu - \frac{E-\mu}{T}\nabla T \Big) \Big\} d\tau'$$
$$\Rightarrow f(\overrightarrow{k}) = f_0(E_{\overrightarrow{k}}) + \tau(E_{\overrightarrow{k}}) \overrightarrow{v}(\overrightarrow{k}). \Big\{ (-\frac{\partial f_0}{\partial E}) \Big(-e\overrightarrow{\mathcal{E}} - \nabla\mu - \frac{E-\mu}{T}\nabla T \Big) \Big\} \tag{17}$$

Assuming that the potential and the temperature gradient are applied in the z direction only,

$$f(\overrightarrow{k}) = f_0(E_{\overrightarrow{k}}) + \tau(E_{\overrightarrow{k}}) v_z(\overrightarrow{k}) \Big\{ (-\frac{\partial f_0}{\partial E}) \Big(-e\overrightarrow{\mathcal{E}_z} - \frac{\partial \mu(z)}{\partial z} - \frac{E-\mu(z)}{T(z)} \frac{\partial T(z)}{\partial z} \Big) \Big\} \tag{18}$$

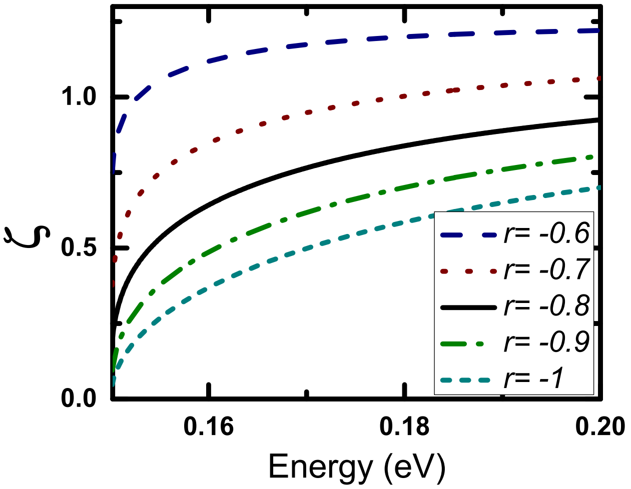

Supplementary Figure 1. Plot of the factor $\zeta = \frac{(E^{\frac{3}{2}} - E_b^{\frac{3}{2}})E^r}{\{(E-E_b)^{r+\frac{3}{2}}\}}$ for various values of r at $E_b = 0.15eV$.

The current density in the z-direction, is therefore, given by:

$$j_z = -e \int \frac{d\overrightarrow{k}}{4\pi^3} v_z(\overrightarrow{k}) f(\overrightarrow{k})$$

$$= -e \int \frac{d\overrightarrow{k}}{4\pi^3} v_z(\overrightarrow{k}) \Big[f_0(E_{\overrightarrow{k}}) + \tau(E_{\overrightarrow{k}}) v_z(\overrightarrow{k}) \Big\{ (-\frac{\partial f_0}{\partial E}) \Big(-e\overrightarrow{\mathcal{E}_z} - \frac{\partial \mu(z)}{\partial z} - \frac{E-\mu(z)}{T(z)} \frac{\partial T(z)}{\partial z} \Big) \Big\} \Big] \tag{19}$$

The integral of the term $v_z(\overrightarrow{k}) f_0(E_{\overrightarrow{k}})$ vanishes since f_0 depends only on energy and is symmetrical in $\overrightarrow{k}$ space.

$$j_z = -e \int \frac{d\overrightarrow{k}}{4\pi^3} \tau(E_{\overrightarrow{k}}) \|\overrightarrow{v_z}(\overrightarrow{k})\|^2 \Big\{ (-\frac{\partial f_0}{\partial E}) \Big(-e\overrightarrow{\mathcal{E}_z} - \frac{\partial \mu(z)}{\partial z} - \frac{E-\mu(z)}{T(z)} \frac{\partial T(z)}{\partial z} \Big) \Big\} \tag{20}$$

$\tau(E_{\overrightarrow{k}})$ and $\|\overrightarrow{v_z}(\overrightarrow{k})\|^2$ depend on $\overrightarrow{k}$ only through the energy $E_{\overrightarrow{k}}$. We can simplify (20) to transform $\overrightarrow{k}$ dependence to energy (E) dependence:

$$j_z = -e \int \tau(E) \|\overrightarrow{v_z}(E)\|^2 D(E) \Big\{ (-\frac{\partial f_0}{\partial E}) \Big(-e\overrightarrow{\mathcal{E}_z} - \frac{\partial \mu(z)}{\partial z} - \frac{E-\mu(z)}{T(z)} \frac{\partial T(z)}{\partial z} \Big) \Big\} dE \tag{21}$$

The term within the second bracket is the driving force for the current and the term $\tau(E)\|\overrightarrow{v_z}(E)\|^2 D(E)$ defines the ease with which the driving force can cause a flow of the

current. An ideal energy filter should block the the current flow from the cold contact to the hot contact due to the negative part of the driving force and therefore only filter out the current produced due to the positive part of the driving force. For the same applied voltage and temperature gradient and assuming an ideal energy filter, the overall current should be given by the equation:

$$j_z = -e\int_{\epsilon}^{\infty} \tau(E)\|\overrightarrow{v_z}(E)\|^2 D(E)\Big\{(-\frac{\partial f_0}{\partial E})\Big(-e\overrightarrow{\mathcal{E}_z} - \frac{\partial \mu(z)}{\partial z} - \frac{E-\mu(z)}{T(z)}\frac{\partial T(z)}{\partial z}\Big)\Big\}dE, \quad (22)$$

where ϵ is defined by the equation:

$$\Big(-e\overrightarrow{\mathcal{E}_z} - \frac{\partial \mu(z)}{\partial z} - \frac{\epsilon-\mu(z)}{T(z)}\frac{\partial T(z)}{\partial z}\Big) = 0.$$

The generated power would increase with increase in the current. In case of an ideal filter, a sufficient but not necessary condition for improvement of generated power with filtering is that $\tau(E)\|\overrightarrow{v_z}(E)\|^2 D(E)$ is an increasing function of E. In other words,

$$\frac{\tau(E+E_b)\|\overrightarrow{v_z}(E+E_b)\|^2 D(E+E_b)}{\tau(E)\|\overrightarrow{v_z}(E)\|^2 D(E)} > 1, \quad (23)$$

for $E_b > 0$. Here E_b is the cut-off energy for filtering. For isotropic and local scattering processes, $\tau(E)$ can generally be approximated as $\tau(E) = \sum_i k_i E^{r_i}$. In case of single moded nanowires, $\|\overrightarrow{v_z}(E)\|^2 D(E) = 2\sqrt{\frac{2\pi E}{m_l h^2}}$. The minimum value of r for which energy filtering can enhance the generated power in case of perfect filtering is therefore $r > r_{min} = -\frac{1}{2}$. For imperfect filtering, the value of r_{min} may further increase.

For bulk generators, a direct calculation of $\Upsilon(E) = \tau(E)\sum_m v_z^2(E-E_m)D_{1D}(E-E_m)$ is non-trivial due to the presence of coupling between transverse electronic modes. The value of $D(E)$ contributing to conduction cannot be defined properly in this case due to partial momentum conservation. However assuming uncoupled mode transport, we can draw an upper limit on the value of r_{min}. It can be shown that for perfect filtering and no effect of scattering near the barrier on the performance of the device, an assumption of uncoupled modes in electron transport gives:

$$\begin{aligned} < v_z^2(E)D(E) > &= \sum_m v_z^2(E-E_m)D_{1D}(E-E_m) \\ &= \int_0^{(E-E_b)} 2\frac{(E-E_m)}{m_l}\sqrt{\frac{2\pi m_l}{h^2}}\frac{1}{\sqrt{E-E_m}}\Big(\frac{4\pi m_t}{h^2}dE_m\Big) \\ &= \frac{16\pi}{3}\frac{1}{h^3}\sqrt{\frac{2\pi m_t^2}{m_l}}(E^{\frac{3}{2}} - E_b^{\frac{3}{2}}), \end{aligned} \quad (24)$$

for $E > E_b$. Here $<>$ denotes the average value of the argument and m denotes all possible modes that are available for conduction. Assuming $\tau(E) = k_o E^r$, (23) translates to:

$$\zeta = \frac{(E^{\frac{3}{2}} - E_b^{\frac{3}{2}})E^r}{\{(E - E_b)^{r+\frac{3}{2}}\}} > 1$$

It can be shown that for $E_b = 0.15eV$, the above condition is valid for $r \gtrapprox -0.7$ (See Fig. 1).

IV. INTERMODE CURRENT PROFILE FOR HIGHER ORDER SCATTERING MECHANISMS.

We plot in Fig. 2, the profiles of I_{Total}, I_{Direct} and $I_{Intermode}$ at the maximum power at various voltage biases for higher order scattering mechanisms in case of both Approach A and Approach B. In these cases, I_{Direct} in Approach B decreases when compared with Approach A. Such a decrease in Approach B is however compensated to a large extent due to the increase in $I_{Intermode}$. With decrease in the value of r beyond a certain point, the decrease in I_{Direct} with energy filtering becomes more compared to the increase of $I_{Intermode}$, resulting in an overall decrease in I_{Total} in the case of Approach B compared to Approach A (demonstrated in bottom panel of Fig. 2).

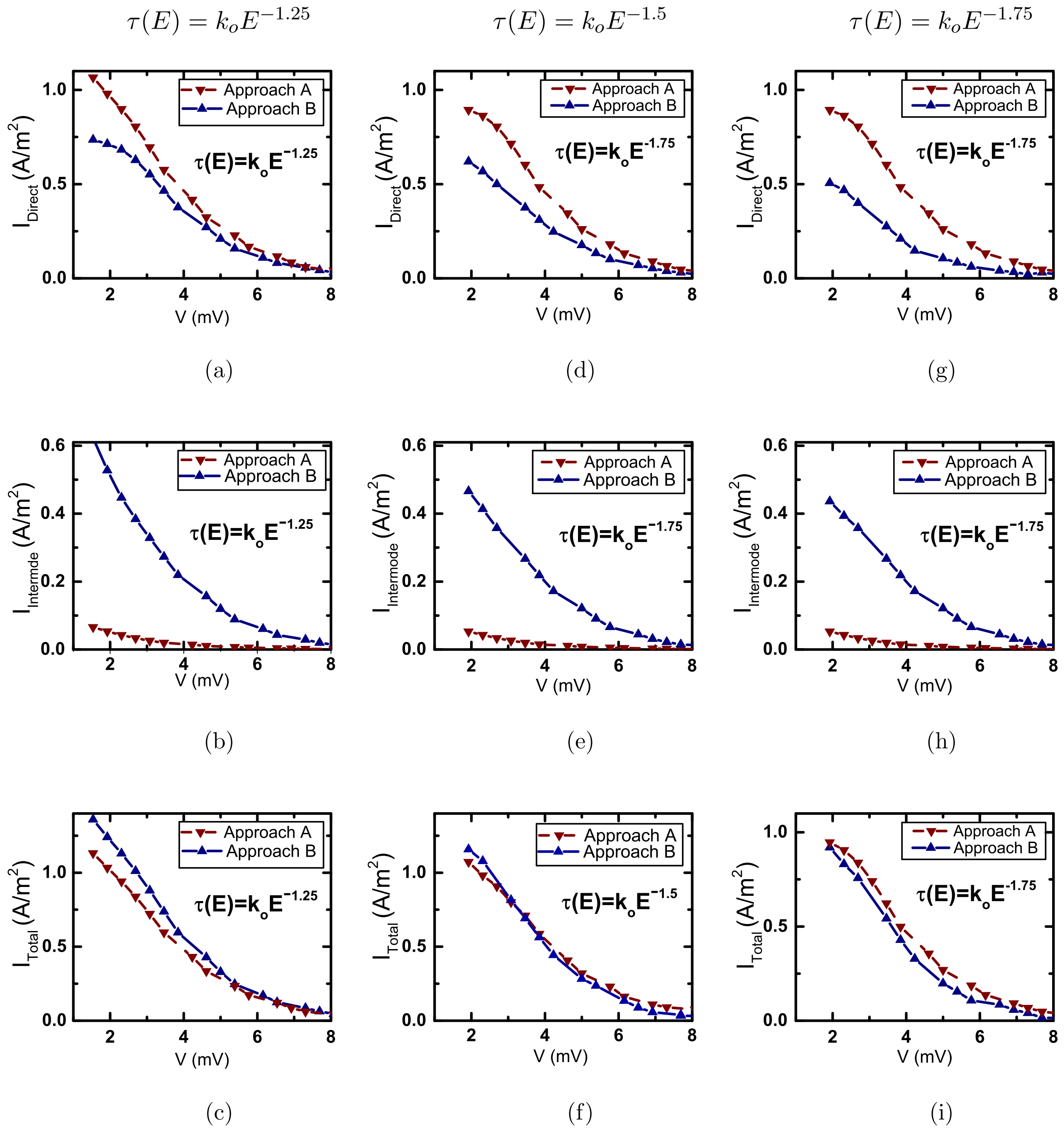

Supplementary Figure 2. Plot of different current profiles at the maximum power at different voltage biases for Approach A and Approach B. *Top panel*: Plot of I_{Direct}. *Middle panel*: Plot of $I_{Intermode}$ *Bottom panel*: Plot of I_{Total} Simulations done for $r = -1.25$ (*left panel*), $r = -1.5$ *(middle panel)* and $r = -1.75$ *(right panel)*. Simulations in case of Approach B are done for a $20nm$ long device with an embedded Gaussian energy barrier ($\sigma_w = 2.7nm,\ E_b = 150meV$)

[1] N.W. Ashcroft and N.D. Mermin. *Solid State Physics*. Saunders College, Philadelphia, 1976.

[2] Supriyo Datta. *Electronic Transport in Mesoscopic Systems.* Cambridge University Press, May 1997.

[3] Supriyo Datta. *Quantum Transport:Atom to Transistor.* Cambridge Press, 2005.

[4] Supriyo Datta. *Lessons from nanoelectronics: a new perspective on transport.* Lessons from nanosciences: A lecture note series. World Scientific, Singapore, 2012.

[5] Luca Selmi David Esseni, Pierpaolo Palestri. *Nanoscale MOS Transistors Semi-Classical Transport and Applications.* Cambridge, 2011.

[6] Mark Lundstrom. *Fundamentals of Carrier Transport.* Cambridge University Press, second edition, 2000.

[7] Aniket Singha, Subhendra D. Mahanti, and Bhaskaran Muralidharan. Exploring packaging strategies of nano-embedded thermoelectric generators. *AIP Advances*, 5(10), 2015.
